# Elevated Expression of PDZD11 Is Associated With Poor Prognosis and Immune Infiltrates in Hepatocellular Carcinoma

**DOI:** 10.3389/fgene.2021.669928

**Published:** 2021-05-21

**Authors:** Yao Chen, Haifeng Xie, Ting Xie, Xunjun Yang, Yilin Pang, SongDao Ye

**Affiliations:** ^1^Department of Pathology, The First Affiliated Hospital of Wenzhou Medical University, Wenzhou, China; ^2^Hangzhou Traditional Chinese Medicine (TCM) Hospital Affiliated to Zhejiang Chinese Medical University, Hangzhou, China; ^3^Zhejiang Provincial Key Laboratory of Medical Genetics, Key Laboratory of Laboratory Medicine, Ministry of Education, School of Laboratory Medicine and Life Sciences, Wenzhou Medical University, Wenzhou, China; ^4^Department of Laboratory Medicine, The Second Affiliated Hospital and Yuying Children’s Hospital of Wenzhou Medical University, Wenzhou, China

**Keywords:** PDZD11, hepatocellular carcinoma, prognostic biomarker, immune infiltrates, functional network analysis

## Abstract

Epithelial cells are held together by tight and adherent junctions, which are destroyed by the activation of epithelial-to-mesenchymal transition (EMT). The PLEKHA7-PDZD11 complex has been reported to be important for epithelial cell adhesion and connecting tissues. However, there is no research regarding the expression and role of PDZD11 in liver hepatocellular carcinoma (LIHC) progression. Here, we analyzed *PDZD11* mRNA expression and its clinical results in LIHC patient RNA sequencing data based on different open databases. Furthermore, we examined differences in PDZD11 expression in LIHC tissues and cell lines using western blotting and real-time qPCR. These results are the first to report that the mRNA and protein levels of PDZD11 are significantly overexpressed in LIHC. Moreover, high expression of *PDZD11* was correlated with poor overall survival in patients with LIHC. Gene regulatory network analysis suggested that PDZD11 is mainly involved in copper ion homeostasis, proteasome, and oxidative phosphorylation pathways. Interestingly, we found that PDZD11 levels were positively correlated with the abundance of immune infiltrates. In particular, higher infiltration levels of CD4^+^ T cells and macrophage subsets significantly affected LIHC patient prognosis. Taken together, these results demonstrate that PDZD11 could be a potential diagnostic and prognostic biomarker in LIHC.

## Introduction

Liver hepatocellular carcinoma (LIHC) accounts for the most common form of primary liver cancers (Villanueva, 2019), with an increasing incidence, particularly in East Asia (Bray et al., 2018; Siegel et al., 2019). LIHC is currently the third leading cause of cancer-related death worldwide (Jiang et al., 2019). LIHC likely occurs in patients with underlying liver diseases since infection with the hepatitis B or C virus (HBV or HCV) and long-term intoxication with alcohol or aflatoxin are the leading risk factors for developing LIHC (Jemal et al., 2017; Villanueva, 2019). Due to the high rate of recurrence and metastasis, the 5-year overall rate of survival for LIHC is only 18%, making liver cancer the second-leading cause of cancer deaths, after pancreatic cancer (Jemal et al., 2017). Although operative treatment may be effective in the early stage of LIHC, the 5-year survival rate after developing to later stage is only 50–70% [European Association for the Study of the Liver (EASL), 2018]. Therefore, it is important to further screen LIHC oncogenes to help identifying novel biomarkers, therapeutic targets and immune-related biomarkers, and ultimately contribute to better diagnosis and prognosis of LIHC.

The human *PDZD11* gene is located at chromosome Xq13.1 and is 3.92 kb long with 6 exons. The PDZD11 protein (140 aa) is a ubiquitously expressed small protein and mainly composed of a single PDZ domain (Stephenson et al., 2005). Previous studies have shown that diseases associated with PDZD11 include Purulent Acute Otitis Media and Middle Ear Disease (GeneCards database). PDZD11 was previously known as PISP, based on its interaction with the plasma membrane calcium ATPase (PMCA) b-splice variants, which may play a role in their sorting to or from the plasma membrane (Goellner et al., 2003). PDZD11 is also known as AIPP1, because it interacts with Menkes copper ATPase (ATP7A), which involves in maintaining copper homeostasis (Stephenson et al., 2005). Nabokina et al. (2011) demonstrated that PDZD11 interacted with human sodium-dependent multivitamin transporter (hSMVT) in intestinal epithelial cells and that this interaction affected biotin uptake process. Shah et al. (2016) reported that the interaction of the N-terminal region of PDZD11 with the WW1 domain of pleckstrin homology domain-containing A7 (PLEKHA7) was essential to stabilize junctional nectins at adherens junctions (AJ), and promote efficient junction assembly. Recent work has also shown that cooperative binding of the tandem WW domains (e.g., WW1 and WW2) of PLEKHA7 to PDZD11 promoted the binding of the C-terminus of Tspan33 to PLEKHA7. Furthermore, the complex formation of PLEKHA7, PDZD11, ADAM10 and its molecular chaperone Tspan33 through promoting the junctional clustering of the α-toxin receptor ADAM10 makes cells more sensitive to the cytotoxic effects of *Staphylococcus aureus* α-toxin (Vasileva et al., 2017; Rouaud et al., 2020).

Epithelial-to-mesenchymal transition (EMT) is a reversible cellular procedure that can transiently dedifferentiate epithelial cells into a mesenchymal phenotype (Dongre and Weinberg, 2019). Epithelial cells build strong connections with their neighbors and an apical-to-basal polarity via the sequential arrangement of adherens junctions, desmosomes, and tight junctions (Thiery et al., 2009). Conversely, EMT confers cells with invasive and metastatic potential, induces stem cell properties, inhibits apoptosis and senescence, and contributes to immunosuppression (Thiery et al., 2009). Therefore, EMT plays a crucial role in embryogenesis, wound-healing, organ fibrosis, tumor invasion and metastasis (Yan et al., 2018). In particular, about 90% of cancer-associated mortality is attributed to metastasis (Chaffer and Weinberg, 2011). Previous studies have shown that the combination of metastasis-related gene signatures and serum alpha-fetoprotein can be used as a good predictor of LIHC prognosis regardless of etiology and race (Yan et al., 2018).

The tumor microenvironment is composed of infiltrating inflammatory cells, stromal cells, and inflammatory mediators (Yan et al., 2018). Undoubtedly, the inflammatory microenvironment associated with hepatitis virus infection is an important factor influencing the invasion and metastasis of LIHC (Yan et al., 2018). Lara-Pezzi et al. (2001) also reported that hepatitis B virus HBx protein was able to induce adherens junction disruption in a src-dependent manner, which might contribute to the development of LIHC.

In this study, we first performed a bioinformatics analysis using different open databases to acquire detailed information about potential functions and prognostic value of PDZD11 in LIHC, and to explore whether the abnormal expression of PDZD11 is closely related to immune infiltrates of LIHC. Further, we verified the expression of PDZD11 in LIHC tissues, various human liver cancer cell lines and matched normal hepatocytes. The findings of this study may help us to understand the role of PDZD11 in the development of LIHC.

## Materials and Methods

### Patients and LIHC Tissue Specimens

Seven pairs of matched LIHC tumor tissues and adjacent normal tissues of each pair of patients were immediately quenched in liquid nitrogen after surgical removal in the First Affiliated Hospital of Wenzhou Medical University. All the patients were clinically and pathologically confirmed as liver cancer. Informed consent was approved by the board of directors and the ethics committee of the First Affiliated Hospital of Wenzhou Medical University. Written informed consent was obtained from all subjects.

### Cell Culture

HCCLM3, MHCC97H, HepG2, and L02 cells were cultured in high-glucose DMEM (GIBCO, Waltham, MA, United States) containing 10% FBS (fetal bovine serum) (GIBCO, United States) and antibiotics (100 U/ml penicillin and 100 μg/ml streptomycin) (GIBCO, United States), and incubated in an incubator containing 5% CO_2_ at 37°C.

### Western Blot Analysis

Proteins in clinical tissues and whole-cells were extracted with 1%Triton X-100 lysis buffer supplemented with protease and phosphatase inhibitors (Sigma-Aldrich). Protein concentrations of the extracts were determined by the BCA assay kit (Thermo Fisher Scientific, Waltham, MA, United States). 40 μg of total protein in each sample was separated by a 12% SDS-PAGE gels and transferred onto PVDF membrane (Bio-Rad, Hercules, CA) with a wet transfer system (Bio-Rad, United States). Block the blot in blocking buffer (5% skim milk in TBST) on a shaker at room temperature for 1 h, and then incubated with primary antibodies specific for PDZD11 (ab121210) (Abcam, Cambridge, MA) (1:2,000) and β-actin (Beyotime Biotechnology Co., Ltd., Shanghai, China) (1:5,000) overnight at 4°C. The membrane was washed in TBST for 3 × 15 min and then incubated with horseradish peroxidase (HRP)-conjugated anti-rabbit (1:5,000) and anti-mouse (1:20,000) immunoglobulin G on a shaker at room temperature for 1.5 h. Immunoreactive proteins were visualized using ECL reagent according to the manufacturer‘s protocol (Thermo Fisher Scientific, Rockford, IL). The optical density was quantified by executing ImageJ software.

### Quantitative RT-PCR

LIHC cell lines and hepatocytes L-02 were seeded in 10 cm culture dish at a density of 2 × 10^6^ cells per culture dish. After 36 h of incubation at 37°C, cells were harvested and washed once with ice-cold PBS. The mRNA expression levels of genes were tested by SYBR green-based real-time quantitative PCR. Total RNA was extracted from all the cells using TRIzol reagent (Thermo Fisher Scientific, Waltham, MA, United States) according to the manufacturer‘s instructions. Total RNA (1 μg) was reverse-transcribed into cDNA (+ gDNA wiper) HiScript II Q Select RT SuperMix (Vazyme Biotech Co., Ltd., Nanjing, China) according to the manufacturer‘s instructions. The RT reaction was subsequently used as a template for real-time PCR. The reactions were performed on a CFX Connect^TM^ Real-Time PCR Detection System (Bio-Rad, Hercules, CA) using ChamQ Universal SYBR qPCR Master Mix (Vazyme Biotech Co., Ltd., Nanjing, China). Primer sequences were as follows: PDZD11 5′CGGTGGTTTTCTTGCCTGCC3′ (forward), 5′-TCAGTGTGATGGTTCGGGGC-3′ (reverse) and β-actin 5′-AGCACAGAGCCTCGCCTTTG-3′ (forward), 5′-AAGCCGGCCTTGCACATG-3′ (reverse). The PCR amplification procedures were as follows: pre-denaturation at 95°C for 3 min, followed by 40 cycles of (95°C for 10 s, 60°C for 30 s). Record the threshold cycle number (Ct) for each reaction. The Ct values of target genes were normalized to that of β-actin. Each sample was analyzed in triplicate and repeated 3 times.

### GEPIA2 Database

The expression of *PDZD11* mRNA in LIHC was analyzed using the GEPIA2 database^1^, which was developed by Peking University, China, and is based on The Cancer Genome Atlas (TCGA) and Genotype-Tissue Expression (GTEx) databases, including RNA sequencing and expression data from 33 malignant tumors, 8,587 normal tissues, and 9,736 tumor samples (Tang et al., 2017).

### Oncomine Analysis

The Oncomine v.4.5 database^2^ is a comprehensive and user-friendly online cancer microarray database for DNA and RNA sequence analysis (Rhodes et al., 2007). In our study, mRNA expression levels and DNA copy number of *PDZD11* in normal controls and cancer specimens were obtained from the Oncomine database. The retrieval conditions were as follows: analysis type/cancer vs. normal analysis, cancer type/liver cancer, dataset filters/data type/mRNA or DNA, and sample filters/sample type/clinical specification. The significance threshold was designed using the following specific parameters: *p*-value of 1E-4, -fold change of 2, and gene rank in the top 10%. Student’s *t*-test was used to analyze differences in the expression of *PDZD11* between normal controls and cancer specimens.

### DriverDBV3 Database

DriverDBV3^3^ uses a variety of -omics techniques to identify cancer driver genes and to present them with different molecular features, including somatic mutations, RNA expression, miRNA expression, DNA methylation, copy number variation, and clinical data, in addition to annotation of bases (Liu S.H. et al., 2020). The Gene Summary of *PDZD11* in various cancer tissues and mRNA expression of *PDZD11* in LIHC was analyzed using the DriverDBV3 database. Survival with a log-rank *p* < 0.05, was considered statistically significant.

### UALCAN Database Analysis

The UALCAN database^4^ is a website for online analysis based on level 3 RNA-seq and clinical data of 31 cancer types from TCGA datasets (Chandrashekar et al., 2017). We used this database to analyze the differential expression and promoter methylation profile of *PDZD11* in primary LIHC tissues and their association with clinicopathological parameters. Student’s *t*-test was used to generate *p*-values; after Bonferroni correction for multiple measures, *p* was still < 0.05, which was statistically significant.

### cBioPortal Analysis

The cBioPortal^5^ is an open-access web resource that provides visualization and analysis of multidimensional cancer genomics data (Gao et al., 2013). In this study, genetic alterations to *PDZD11* in LIHC patients (TCGA, Firehose Legacy, 360 patients/samples) were investigated using the cBioPortal database.

### Protein-Protein Interaction (PPI) Network Analysis

PPI network analysis of PDZD11 was conducted using the STRING^6^ (von Mering et al., 2003) and GeneMANIA^7^ (Warde-Farley et al., 2010) online databases. We also used GeneMANIA to construct gene networks and predict the biological functions of gene sets in which Gene Set Enrichment Analysis (GSEA) was identified as being enriched in LIHC.

### LinkedOmics Database Analysis

The LinkedOmics database^8^ (Vasaikar et al., 2018) is an online open-access powerful bioinformatics platform, which includes multi-omics information and clinical data involving 11,158 patients and 32 cancer types in the TCGA project. LinkedOmics was used to study genes differentially expressed in correlation with *PDZD11* in LIHC. Pearson’s correlation coefficient was applied to statistical analysis of the results produced by LinkedOmics. Then, genes positively and negatively correlated with *PDZD11* in LIHC were selected based on the criteria of coefficient > 0.3 and <−0.3. Finally, we enriched these gene sets by Gene Ontology (GO) analysis and Kyoto Encyclopedia of Genes and Genomes (KEGG) pathway enrichment analysis using the DAVID database^9^ (Huang da et al., 2009), and the results were visualized using an online platform^10^. Moreover, GSEA was utilized to perform various enrichment analyses, including for kinase targets, miRNA targets, and transcription factor targets. Ranking was based on the criteria of false discovery rate (FDR) <0.05, and 500 simulations were performed.

### TIMER Analysis

Tumor Immune Estimation Resource (TIMER)^11^ is a comprehensive website for the systematic analysis of tumor-infiltrating immune cells (Li et al., 2017). TIMER2.0^12^ is the latest version of TIMER. We first analyzed the expression of *PDZD11* in various tumors using the TIMER database, and the results were analyzed statistically using Wilcoxon rank sum test. Then, correlations between the expression of PDZD11 and the abundance of the six immune cell types (B cells, CD8^+^ T cells, CD4^+^ T cells, macrophages, neutrophils, and dendritic cells) in LIHC were analyzed using Spearman tests (tumor purity adjusted). Finally, the survival module was used to draw Kaplan-Meier plots for immune infiltrates and *PDZD11* to determine survival differences. Statistical significance was set at *p* < 0.05.

### Statistical Analysis

GraphPad Prism (v.5.0) for Windows was used for statistical analysis, and *p* < 0.05 was considered statistically significant. The log-rank test was used in Kaplan-Meier survival analysis. Student’s *t*-test and Wilcoxon rank sum test were employed in two-group comparisons. Moreover, we conducted Bonferroni’s correction for multiple measurements to ensure the credibility of multiple group comparisons. After Bonferroni correction, *p* was still less than 0.05, which represents a statistically significant difference.

## Results

### Elevated Expression of PDZD11 in LIHC

**FIGURE 1:**
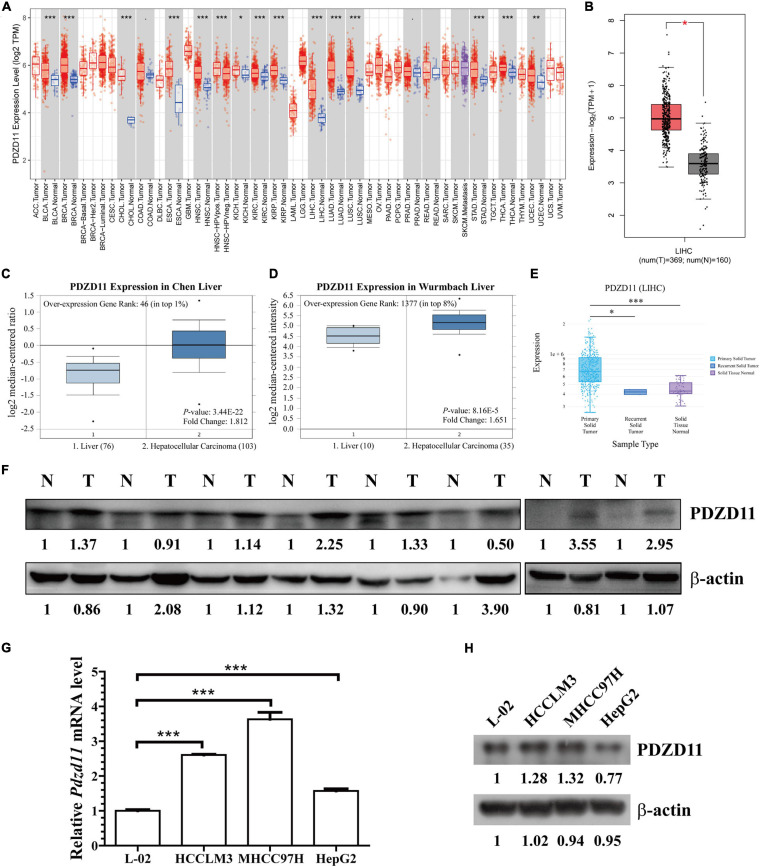
PDZD11 expression levels in LIHC. **(A)** Transcription levels of *PDZD11* in different types of cancers (TIMER database). **(B)** *PDZD11* mRNA expression levels in LIHC tissues and adjacent normal liver tissues from GEPIA 2 database. **(C,D)** Box plots show *PDZD11* mRNA expression in liver (left plot) and hepatocellular carcinoma tissue (right plot) of the Chen Liver **(C)** and Wurmbach Liver **(D)** datasets. The fold-change of *PDZD11* expression in LIHC was determined using the Oncomine database. The threshold was designed using the following specific parameters: *p* = 1E-4, fold change = 2, and gene rank 10%. **(E)** mRNA expression of *PDZD11* in primary solid tumors, recurrent solid tumors, and adjacent normal liver tissues (DriverDBV3 database). **(F)** A representative western blot showing PDZD11 protein is expressed in LIHC tissues (T) and matched normal liver tissues (N) (*n* = 8). **(G,H)** Real-time qPCR and Western blotting analysis of *PDZD11* mRNA **(G)** and protein expression **(H)** in human hepatocytes and LIHC cell lines. *p < 0.05; **p < 0.01; ***p < 0.001.

To determine the differential expression of PDZD11 in diverse cancer types, *PDZD11* mRNA expression was analyzed using the TIMER database. It was shown that the mRNA level of *PDZD11* was significantly upregulated in bladder, breast, gallbladder, esophagus, kidney, liver, lung, gastric, thyroid, and uterine corpus endometrial carcinoma (Figure 1A). Further analysis showed that *PDZD11* was overexpressed in LIHC patients in the GEPIA2 database (Figure 1B). In the ONCOMINE database, *PDZD11* was also identified with significantly higher levels in LIHC in multiple datasets. In the Chen Liver dataset, *PDZD11* overexpression was found in LIHC tissues compared with normal tissues with a -fold change of 1.812 (*p* = 3.44E-22), while Wurmbach observed a 1.651-fold increase in *PDZD11* mRNA expression in LIHC samples (*p* = 8.16E-5) (Figures 1C,D). In addition, we analyzed *PDZD11* expression using the DriverDBV3 database. We found that the results were largely consistent with those in the ONCOMINE database. However, there was no statistically significant difference in the mRNA expression of *PDZD11* observed in recurrent solid tumors compared to adjacent normal liver tissues (Figure 1E). Consistently, protein analysis involving eight patients (including eight tumor tissue and eight matched adjacent normal tissues) diagnosed with liver cancer confirmed that *PDZD11* abundance was elevated in LIHC tissues (Figure 1F). Additionally, we found increased levels of *PDZD11* in LIHC cell lines at both the mRNA and protein levels (Figures 1G,H) compared to that found in normal human L-02 hepatocytes. However, the protein expression of PDZD11 in HepG2 cells was significantly lower than that in L-02 cells.

### Relationship Between *PDZD11* mRNA Levels and Clinicopathological Parameters in LIHC Patients

**FIGURE 2:**
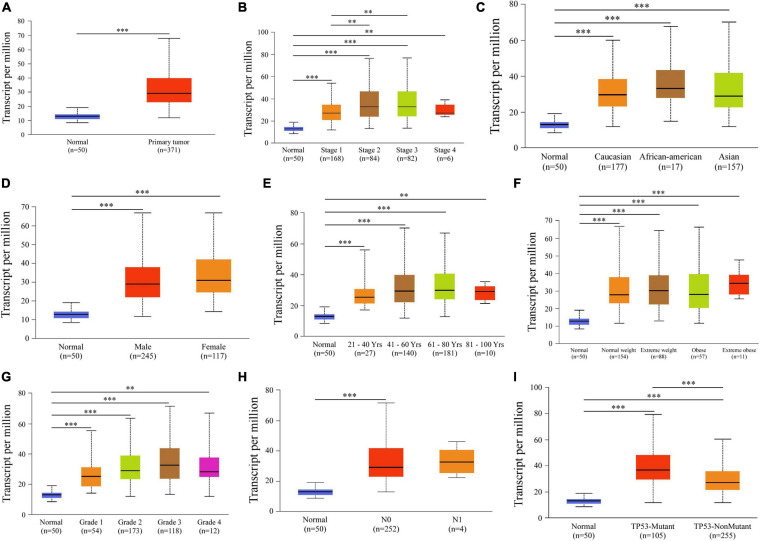
Analysis of subgroup expression of *PDZD11* in LIHC (UALCAN database). **(A)** *PDZD11* mRNA expression in LIHC tissue and adjacent normal liver tissue. **(B–I**) Box plot shows the *PDZD11* mRNA expression of LIHC patients in the subgroups of different cancer stages **(B)**, race **(C)**, gender **(D)**, age **(E)**, weight **(F)**, tumor grade **(G)**, nodal metastasis status **(H),** and TP53 mutation status **(I)**. The data are shown as mean ± SE. ^∗^*p* < 0.05; ^∗∗^*p* < 0.01; ^∗∗∗^*p* < 0.001. The asterisk indicates a significant difference between the two sets of data.

Next, relationships between *PDZD11* mRNA expression and clinicopathological parameters of LIHC patients were analyzed using the UALCAN database. The results showed that *PDZD11* was upregulated in primary LIHC tissues compared to adjacent normal tissues (Figure 2A, *p* < 0.001). As shown in Figures 2B–I, according to subgroup analysis based on race, gender, age, weight, and lymph node metastasis status, the mRNA expression of *PDZD11* in LIHC patients was evidently higher than that in healthy individuals. In particular, the expression of *PDZD11* mRNA was clearly correlated with more advanced and less-differentiated tumors in LIHC patients, who tended to express higher *PDZD11* mRNA levels. The highest mRNA expression of *PDZD11* was found in stage 3 and/or tumor grade 3 cases (Figures 2B,G). The reason why mRNA expression of *PDZD11* in stage 3 and/or tumor grade 3 seemed to be higher than that in stage 4 and tumor grade 4 may be due to the small number of samples. In addition, *PDZD11* mRNA expression was positively correlated with *TP53* mutation status, and was also significantly elevated in LIHC patients with *TP53* mutations (Figure 2I).

### Frequency and Types of *PDZD11* Alterations in LIHC

**FIGURE 3:**
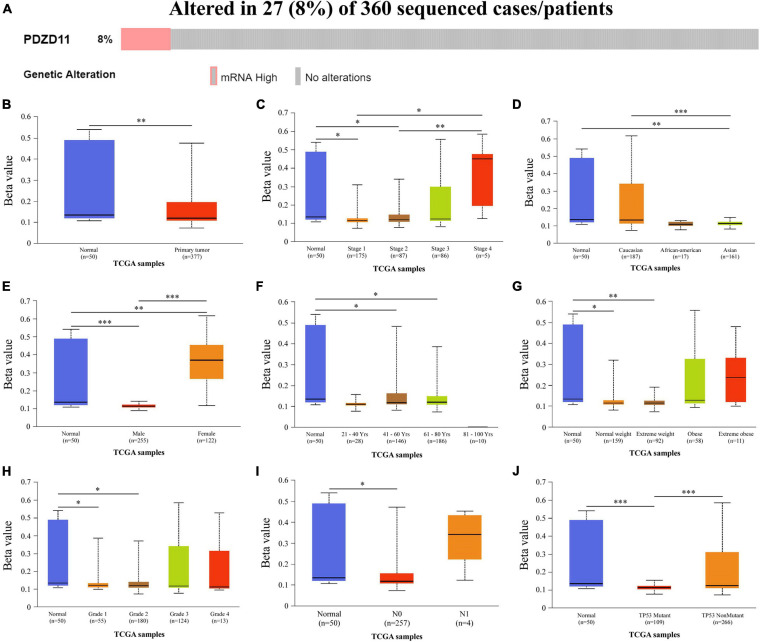
Genetic alternations and promoter methylation levels of *PDZD11* in LIHC. **(A)** Oncoprint in cBioPortal showed the distribution and proportion of samples with alternations in *PDZD11*. **(B)** The promoter methylation levels of *PDZD11* in LIHC. **(C–J)** Box plots show promoter methylation level of *PDZD11* in normal vs. LIHC tissues and different individual cancer stages **(C)**, race **(D)**, gender **(E)**, age **(F)**, weight **(G)**, tumor grade **(H)**, nodal metastasis status **(I)**, and TP53 mutation status **(J)** (UALCAN database). Data are shown as mean ± SE. ^∗^*p* < 0.05; ^∗∗^*p* < 0.01; ^∗∗∗^*p* < 0.001.

Genetic alterations to *PDZD11* in LIHC were evaluated using the cBioPortal database. As shown in Figure 3A, among the 360 LIHC patients sequenced, 27 showed genetic alterations, with a mutation rate of 8%. Moreover, we observed that mRNA upregulation was the only aberrant type of genetic alteration involving *PDZD11* in LIHC.

### Decreased *PDZD11* Promoter Methylation Levels in LIHC

To analyze why expression of *PDZD11* mRNA was significantly higher in LIHC tissues than in adjacent normal liver tissues, we used the UALCAN database to evaluate the extent of *PDZD11* promoter methylation in LIHC samples and investigated the association between promoter DNA methylation and *PDZD11* expression levels. The results indicated that *PDZD11* promoter methylation levels were lower in LIHC cases than in normal control samples (Figure 3B). To explore the factors that affect levels of *PDZD11* promoter methylation, we further analyzed promoter DNA methylation of *PDZD11* in different subgroups according to different clinicopathological parameters. The subgroup analysis results showed that promoter methylation of *PDZD11* was possibly affected by individual cancer stages, race, gender, age, weight, tumor grade, nodal metastasis status, and *TP53* mutation status in LIHC (Figures 3C–J).

### Prognostic Value of *PDZD11* mRNA Expression in LIHC Patients

**FIGURE 4:**
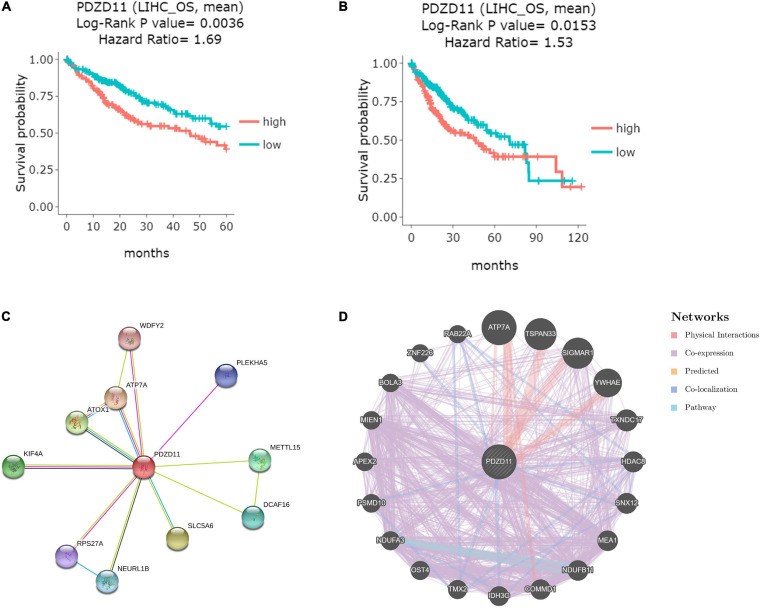
Visual summary of the prognostic value and biological interaction network of PDZD11 in LIHC. **(A,B)** Kaplan-Meier plot of the relationship of *PDZD11* gene expression and survival in LIHC patients (DriverDBV3 database). The LIHC patient samples are stratified into 2 groups using the mean expression value as the cut-off point: 149 samples with highly expressed *PDZD11* mRNA (red) and 216 samples with lowly expressed *PDZD11* mRNA (green). *Y*-axis is survival probability. The left figure is the 5-year survival, and the right figure shows overall survival (OS). The *X*-axis is the months of survival period. Log-Rank *P*-value and Hazard Ration (HR) are provided on the top of plots. **(C)** Protein-protein interaction (PPI) network of PDZD11 (Top 10) (STRING database). **(D)** PPI network (Top 20) of PDZD11 from GeneMANIA database.

To explore whether high expression levels of *PDZD11* are associated with cancer-promoting or tumor suppressor genes, we evaluated the prognostic value of *PDZD11* mRNA expression in patients with LIHC using the DriverDBV3 database. As shown in Figures 4A,B, *PDZD11* overexpression was associated with unfavorable 5-year survival [hazard ratio (HR) = 1.69, log-rank *p* = 0.0036] and overall survival (OS, HR = 1.53, log-rank *p* = 0.0153) in LIHC patients.

### Biological Interaction Network of PDZD11

Using the STRING and GeneMANIA databases, a functional protein interaction network of PDZD11 was constructed to enrich for possible PDZD11-mediated signaling pathways (Figures 4C,D). ATP7A, a transmembrane protein that functions in copper transport across cell membranes, was the only gene that intersected two protein-protein interaction (PPI) networks (Schmidt et al., 2018). Furthermore, STRING was used to perform GO and KEGG analyses to determine the functional enrichment of these 29 interactors. The results indicated that biological processes included copper ion homeostasis and copper ion transmembrane transport; Cellular components analysis found that these proteins are localized mainly in endosomes and early endosomes. Molecular function analysis indicated that these proteins are primarily involved in copper-dependent protein binding, copper ion transmembrane transporter activity, copper ion binding, copper chaperone activity and phosphatidylinositol-3,5-bisphosphate binding (data not shown).

### Enrichment Analysis of PDZD11 Functional Networks in LIHC

#### Predicted Functions and Pathways of Co-expressed Genes Correlate With *PDZD11* in LIHC

**FIGURE 5:**
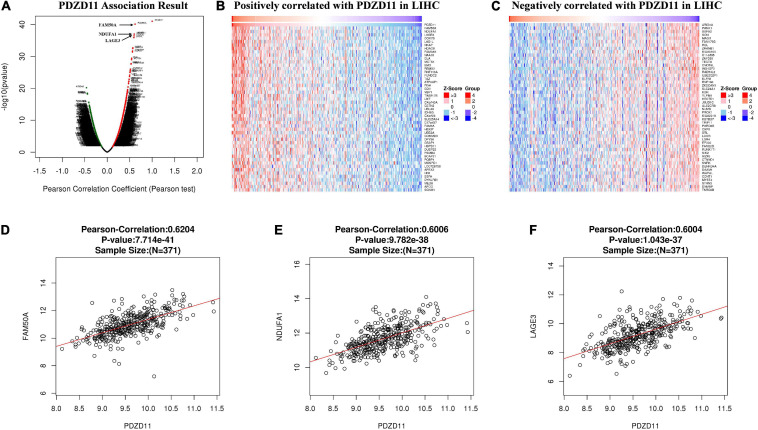
Differentially expressed genes that correlated with *PDZD11* in LIHC (LinkedOmics database). **(A)** A Pearson test was used to determine the correlations between *PDZD11* and differently expressed genes in LIHC. **(B,C)** Heat maps are showing genes (Top 50) positively or negatively correlated with *PDZD11* in LIHC. Red indicates positively correlated genes and blue indicates negatively correlated genes. **(D–F)** The scatter plots mean that *PDZD11* expression is positively correlated with the expression of *FAM50A* **(D)**, *NDUFA1* **(E)**, and *LAGE3* **(F)**.

LinkedOmics was used to analyze TCGA mRNA sequencing data from 371 LIHC patients. Pearson’s test was used to analyze the co-expression of genes correlated with *PDZD11* levels in LIHC. As shown in the volcano plot (Figure 5A), 2,960 genes (dark red dots) showed significant positive correlation with *PDZD11*, whereas 3,234 genes (dark green dots) showed opposite correlations (false discovery rate (FDR) < 0.01). The top 50 significant genes were positively and negatively associated with *PDZD11*, as shown in the heat map (Figures 5B,C). As shown in Figures 5D–F, the mRNA expression of *PDZD11* showed the strongest positive association with expression of *FAM50A* (Pearson correlation = 0.62, *p* = 7.71e-41), *NDUFA1* (Pearson correlation = 0.60, *p* = 9.78e-38), and *LAGE3* (Pearson correlation = 0.60, *p* = 1.04e-37), which reflect changes in the spliceosome complex (Lee et al., 2020), mitochondrial respiratory chain complex I (Fernandez-Moreira et al., 2007), and the KEOPS/EKC complex (tRNA modification complex) (Wan et al., 2017).

**FIGURE 6:**
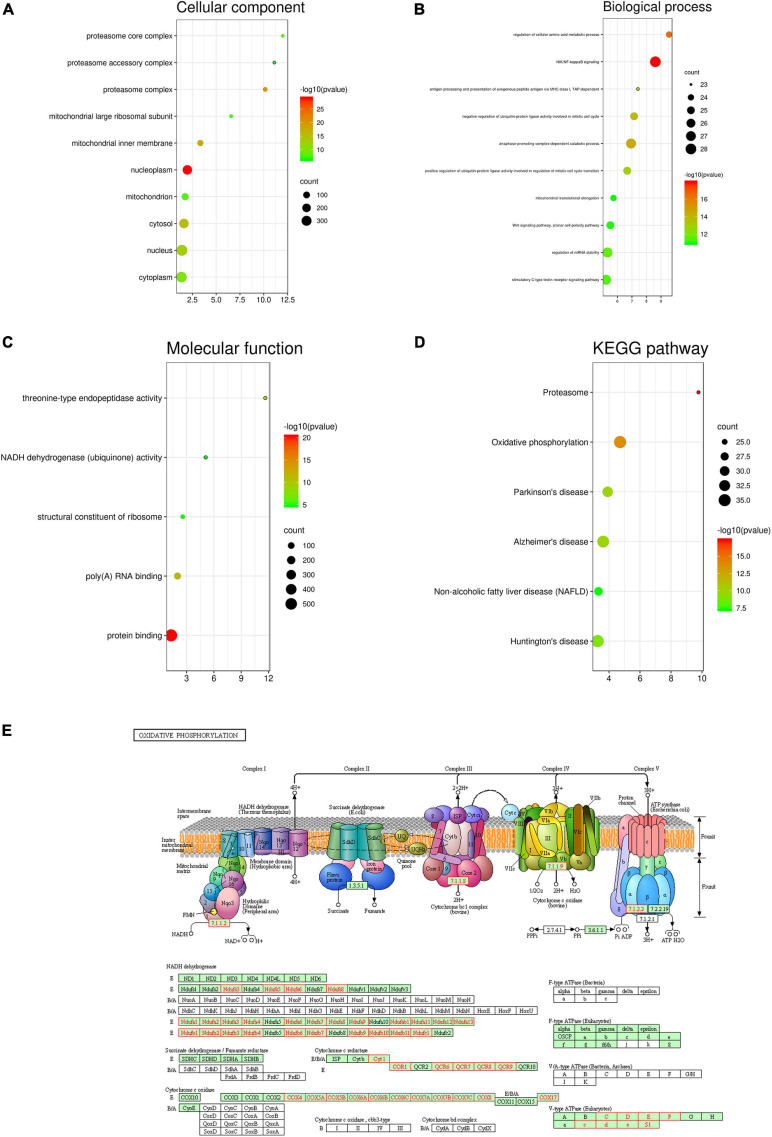
Enrichment analysis of *PDZD11* co-expression genes in LIHC. **(A–D)** The significantly enriched GO annotations and KEGG pathways of *PDZD11* co-expression genes in LIHC are analyzed using DAVID. Based on the Pearson test (Figures 5A–C), we selected the positively and negatively correlated genes with coefficient > 0.3 and < –0.3 (LinkedOmics and bioinformatics databases). **(E)** KEGG pathway annotations of the oxidative phosphorylation pathway. Red marked nodes are associated with the Leading Edge Genes. FDR < 0.05 was considered statistically significant.

Furthermore, based on the results of the Pearson test (Figures 5A–C), we selected positively and negatively correlated genes with coefficients > 0.3 and <−0.3. Finally, 617 genes positively correlated with *PDZD11* and 411 genes negatively correlated with *PDZD11* were selected (FDR < 0.001). Moreover, these genes were used for GO and KEGG enrichment analyses using the DAVID database. The cutoff criterion was set at FDR < 0.01. As shown in Figures 6A–D, cellular component analysis indicated that these proteins were mainly located in the nucleoplasm, proteasome complex, and mitochondrial inner membrane. Biological processes were primarily enriched in NIK/NF-κB signaling, regulation of cellular amino acid metabolic processes, and anaphase-promoting complex-dependent catabolic processes. Molecular function analysis revealed that these proteins were mostly involved in protein binding, poly(A) RNA binding, and threonine-type endopeptidase activity. KEGG pathway results showed that the co-expressed genes for the most part participated in proteasomes, oxidative phosphorylation (OXPHOS) (Figure 6E), and Alzheimer’s disease.

### PDZD11 Networks of Kinase, MicroRNA or Transcription Factor Targets in LIHC

To further explore the gene regulatory network of *PDZD11* in LIHC, we also analyzed the important kinase, miRNA, and transcription factor target networks that were connected to *PDZD11* in LIHC via gene set enrichment analysis (GSEA). The results showed that the most frequent kinase targets, miRNA targets, and transcription factor targets were kinase CDK5, three miR-200 family members (miR-200b, miR-200c, and miR-429), and V$SOX9_B1, respectively (Table 1 and Supplementary Tables 5–7). Furthermore, PPI networks were constructed by STRING, and biological enrichment was performed using the DAVID database, indicating that all three gene sets were mainly involved in the KEGG pathway of prostate cancer, MAPK signaling pathway, and transcriptional dysregulation in cancer (Supplementary Figures 1–3).

**TABLE 1:** Kinase, miRNA and transcription factor-target networks of *PDZD11* in LIHC (LinkedOmics).

Enriched category	Geneset	Leading edge number	FDR	*P-*value
Kinase target	Kinase_CDK5	26	0.0066696	0
	Kinase_NLK	5	0.010671	0
	Kinase_MAPK7	14	0.034682	0
	Kinase_DYRK1A	7	0.045353	0.0040650
miRNA target	GTGTTGA,MIR-505	46	0	0
	CAGTATT,MIR-200B,MIR-200C,MIR-429	155	0	0
	ACTGAAA,MIR-30A-3P,MIR-30E-3P	81	0	0
	AAAGGGA,MIR-204,MIR-211	101	0	0
	TACTTGA,MIR-26A,MIR-26B	131	0	0
Transcription factor target	GGAANCGGAANY_UNKNOWN	35	0	0
	V$FREAC4_01	49	0	0
	V$HOX13_01	15	0	0
	V$SOX9_B1	80	0	0
	V$STAT5A_02	50	0	0

*FDR, false discovery rate from Benjamini and Hochberg from gene set enrichment analysis (GSEA). V$, the annotation found in Molecular Signatures Database (MSigDB) for transcription factors (TF).*

### Association of PDZD11 Expression and Immune Infiltration in LIHC

**FIGURE 7:**
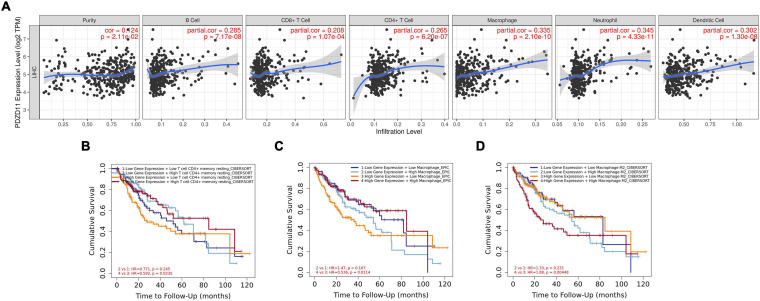
Associations between mRNA expression of *PDZD11* and immune infiltration in LIHC (TIMER2.0 database). **(A)** Association of *PDZD11* expression with abundance of immune infiltrates (B cells, CD8^+^ T cells, CD4^+^ T cells, macrophages, neutrophils and dendritic cells). **(B–D)** The effect of *PDZD11* expression in correlation with infiltration levels of immune cells on the prognosis of LIHC.

LIHC is one of the most common malignant tumors (Okajima et al., 2017). Because *PDZD11* overexpression is associated with poor prognosis in LIHC patients (Figure 4), we explored whether the expression of *PDZD11* was correlated with levels of immune infiltration in LIHC from the TIMER database and/or TIMER2.0 database. As shown in Figure 7, there was a positive correlation between *PDZD11* expression and infiltration by B cells, CD8^+^ T cells, CD4^+^ T cells, macrophages, neutrophils, and dendritic cells. Furthermore, under the premise of high expression of *PDZD11* mRNA in LIHC, we found that higher infiltration levels of two immune cells (T cell CD4 + memory resting-CIBERSORT, and Macrophage-EPIC) were associated with better survival outcomes in LIHC patients (Figures 7B,C). In contrast, higher infiltrating levels of the macrophage M2 subset was a risk factor for disease prognosis in LIHC patients (Figure 7D).

## Discussion

EMT-induced changes in epithelial cell plasticity are evidenced by the loss of epithelial markers, such as the adherence junction component E-cadherin and cytokeratins of the intermediate filament system (K8, K18, K19). Conversely, the expression of mesenchymal proteins such as N-cadherin, α-SMA, FSP-1, and the EMT transcription factors Snail (SNA1), Slug (SNA2), Twist, and ZEB are increased (Giannelli et al., 2016). Konopka et al. (2007) have also reported that junctional adhesion molecule-A (JAM-A) is critical for the formation of pseudocanaliculi and regulates E-cadherin expression through feedback signaling pathways in hepatic cells. However, the present study lacked a well-defined consensus on EMT-MET (mesenchymal-epithelial transition) biomarkers, which hinders definitive conclusions on how EMT affects clinical outcomes in LIHC patients (Giannelli et al., 2016). Therefore, there is an urgent need to identify biomarkers or therapeutic targets related to EMT for early diagnosis and for predicting the progression and recurrence of LIHC.

Current research reports that the interaction of PDZD11 with PLEKHA7 is significantly associated with tight and adherens junctions (Guerrera et al., 2016; Vasileva et al., 2017). However, to the best of our knowledge, no study has investigated the role of PDZD11 in liver cancer. In this study, we provide the first evidence that *PDZD11* mRNA expression is significantly upregulated in LIHC and is associated with poor prognosis (Figures 1A–E, 4A,B). In particular, we demonstrated that *PDZD11* is aberrantly expressed in human liver cancer tissues and cell lines (Figures 1F–H). Moreover, subgroup analysis showed that the mRNA expression of *PDZD11* was also upregulated in different subgroups of LIHC (Figure 2). In particular, the mRNA expression of *PDZD11* increased as tumors progressed (Figures 2B,G). Additionally, we found that the expression of *PDZD11* may be negatively regulated by wild-type p53 at the transcriptional level (Figure 2I). Similarly, previous studies have shown that E-cadherin, the most reliable and closely investigated marker in a large number of LIHC patients, was directly correlated with poorer prognosis and shorter survival (Yamada et al., 2014). Consequently, these results suggest that PDZD11 and the EMT marker E-cadherin could serve as potential diagnostic and prognostic biomarkers in LIHC patients.

mRNA upregulation is the most aberrant type of genetic alteration involving *PDZD11* in LIHC (Figure 3A). We further analyzed *PDZD11* promoter DNA methylation levels and found that the higher expression of *PDZD11* in LIHC may be negatively correlated with the extent of promoter methylation (Figure 3B). Subgroup analysis showed that *PDZD11* promoter methylation level was also downregulated in different subgroups of LIHC (Figures 3C–J). Cano et al. (2000) reported that the expression of the EMT marker E-cadherin is negatively regulated by the transcription factor Snail. These results suggest that the mechanism of high expression of *PDZD11* mRNA in LIHC could be different from the classical Snail/E-cadherin axis.

Further analysis of the gene regulatory network of PDZD11 in LIHC suggested that the functions of these genes were primarily related to copper ion homeostasis, proteasome, and OXPHOS pathway. As shown in Figures 4C,D, *ATP7A* is the only gene that intersects the two PPI networks. A previous study demonstrated that ATP7A is a transmembrane protein that functions in copper transport across cell membranes (Schmidt et al., 2018). Bortezomib is a first-in-class proteasome inhibitor that has been repeatedly demonstrated to exert anti-proliferative, anti-metastatic, and pro-apoptotic effects in LIHC (Yang et al., 2016; Huang et al., 2019). This study showed that the protein expression level of PDZD11 was irreconcilable with its mRNA transcription level. However, this proteasome-mediated PDZD11 protein degradation pathway requires further research. A recent study has also reported that induced E-cadherin expression and subsequent induction of NF-κB signaling increases OXPHOS, glycolysis, and cell proliferation in human gastric adenocarcinomacells (Park et al., 2017). Therefore, further research is needed to determine how abnormal expression of PDZD11 affects OXPHOS in LIHC and its role in LIHC metastasis.

We also sought important networks of target kinases, miRNAs, and transcription factors of the differentially expressed PDZD11 in LIHC. We found that PDZD11 in LIHC was linked to a network of kinases, including CDK5, NLK, and MAPK7. Previous studies have reported that levels of these kinases are significantly higher in human LIHC tissue than in normal liver tissue. Moreover, the downregulated expression of these kinases significantly inhibits the development and growth of LIHC *in vitro* and *in vivo* (Jung et al., 2010; Ehrlich et al., 2015; Lu et al., 2016). The probable miRNAs involved in the regulation of PDZD11 expression in LIHC included miR-505, three miR-200 family members (miR-200b, miR-200c, and miR-429), and two miR-30 family members (miR-30a-3p and miR-30e-3p). Lu et al. (2016) found that miR-505 regulates proliferation, invasion, and EMT in MHCC97 hepatoma cells by targeting high-mobility group box 1 (HMGB1). Ding et al. (2012) showed that the combination of a DNA methyltransferase (DNMT) inhibitor and upregulation of miR-200b could block lung metastasis of mesenchymal-phenotype hepatocellular carcinoma. Wang et al. (2014) indicated that miR-30a-3p inhibits tumor proliferation, invasion, and migration, and is downregulated in LIHC. Our data indicated that V$FREAC4_01, V$HOX13_01, and V$SOX9_B1 may be key transcription factors in the regulation of PDZD11. Liu et al. (2016) demonstrated that Sox9 regulates self-renewal and tumorigenicity by promoting symmetrical cell division of cancer stem cells in LIHC. Taken together, abnormal expression of PDZD11 may modulate tumor cell proliferation, invasion, metastasis, and the development of LIHC by regulating these targets. Further studies are required to verify this hypothesis.

The emergence and development of LIHC are accompanied by a persistent inflammatory reaction. Inflammatory cells in the tumor microenvironment of LIHC mainly include macrophages, infiltrating lymphocytes, neutrophils, mast cells, dendritic cells, and eosinophils (Kim and Bae, 2016; Yan et al., 2018). In particular, Liu W.R. et al. (2020) reported that among these tumor-related regulatory T cells (Tregs), macrophages, and neutrophils are strongly correlated with OS and relapse-free survival (RFS) in LIHC patients. Here, we found that PDZD11 expression in LIHC was positively correlated with infiltrating levels of six immune cell types (i.e., B cells, CD4^+^ T cells, CD8 + T cells, macrophages, neutrophils, and dendritic cells). Moreover, under the premise of high expression of *PDZD11* mRNA in LIHC, the higher infiltration levels of the CD4^+^ memory resting T cell subset were favorable factors for prognosis in LIHC patients. In contrast, the higher infiltration levels of the macrophage M2 subset had an unfavorable prognosis in LIHC (Figure 7). Previous studies have shown that CD4^+^ T cells and tumor-associated macrophages (TAMs) play a central role in pro-tumor immunity; their interactions with tumor cells can directly promote tumor growth, progression, invasion, and metastasis. Conversely, CD8^+^ T cells are responsible for anti-tumor responses, and increased CD8^+^ T cell infiltration usually indicates a better prognosis in LIHC (Yan et al., 2018; Ansari et al., 2020; Li et al., 2020). In summary, these data indicate that PDZD11 is not only a prognostic biomarker, but may also reflect the immune status of LIHC patients.

In summary, these findings highlight the critical role of PDZD11 in the development and progression of LIHC. However, immunohistochemistry and functional analysis are needed in future studies to verify the relationship between PDZD11 and EMT in LIHC at the clinical and cellular levels. In particular, compared with normal human hepatocytes, the overexpression level of *PDZD11* mRNA was significantly higher than its protein level. In addition, the protein expression level of PDZD11 in HepG2 cells was significantly reduced. Therefore, overexpression of *PDZD11* in LIHC could not be ruled out, which is a self-protective feedback regulation mechanism that inhibits tumor metastasis. Further studies are needed to determine whether the aberrant expression of PDZD11 is detrimental or beneficial to patients with LIHC, and further studies are needed to explore how the aberrant expression of PDZD11 regulates the onset and progression of LIHC via EMT and OXPHOS pathways.

## Data Availability Statement

The original contributions presented in the study are included in the article/Supplementary Material, further inquiries can be directed to the corresponding author/s.

## Ethics Statement

The studies involving human participants were reviewed and approved by the board of directors and the ethics committee of the First Affiliated Hospital of Wenzhou Medical University. The patients/participants provided their written informed consent to participate in this study.

## Author Contributions

SY and YP designed and supervised the study. YC and XY collected the patient samples and performed the study. YC, HX, TX, YP, and SY participated in data analysis and figure preparation. YP, SY, and TX revised the article was written. SY and YP reviewed the manuscript. All authors read and approved the final manuscript.

## Conflict of Interest

The authors declare that the research was conducted in the absence of any commercial or financial relationships that could be construed as a potential conflict of interest.

## Footnotes

1 http://gepia2.cancer-pku.cn/#index

2 https://www.oncomine.org/resource/login.html

3 http://driverdb.tms.cmu.edu.tw/

4 http://ualcan.path.uab.edu/

5 https://www.cbioportal.org/

6 https://string-db.org/

7 http://genemania.org/

8 http://www.linkedomics.org/login.php

9 https://david.ncifcrf.gov/home.jsp

10 http://www.bioinformatics.com.cn/

11 https://cistrome.shinyapps.io/timer/

12 http://timer.cistrome.org/

## Supplementary Material

Supplementary Figure 1 Protein-protein interaction (PPI) network of CDK5 Kinase-target networks (STRING).

Supplementary Figure 2 PPI network of MIR-505 (STRING).

Supplementary Figure 3 PPI network of V$FREAC4_01 transcription factor -target networks (STRING).

Supplementary Table 1 Significantly enriched GO annotations (biological processes) of *PDZD11* in LIHC (LinkedOmics).

Supplementary Table 2 Significantly enriched GO annotations (cellular components) of *PDZD11* in LIHC (LinkedOmics).

Supplementary Table 3 Significantly enriched GO annotations (molecular functions) of *PDZD11* in LIHC (LinkedOmics).

Supplementary Table 4 Significantly enriched KEGG pathway annotations of *PDZD11* in LIHC (LinkedOmics).

Supplementary Table 5 Significantly enriched kinase-target networks of *PDZD11* in LIHC (LinkedOmics).

Supplementary Table 6 Significantly enriched miRNA-target networks of *PDZD11* in LIHC (LinkedOmics).

Supplementary Table 7 Significantly enriched transcription factor-target networks of *PDZD11* in LIHC (LinkedOmics).
