# Supplementary material for: Elevated Expression of PDZD11 Is Associated With Poor Prognosis and Immune Infiltrates in Hepatocellular Carcinoma
Source: Front Genet. 2021 May 21;12:669928. doi: 10.3389/fgene.2021.669928 (PMC8176286; doi:10.3389/fgene.2021.669928)
Supplement: Supplementary Figure 1 — Protein-protein interaction (PPI) network of CDK5 Kinase-target networks (STRING). [file Data_Sheet_1.docx]

Supplementary Material

## Supplementary Figures


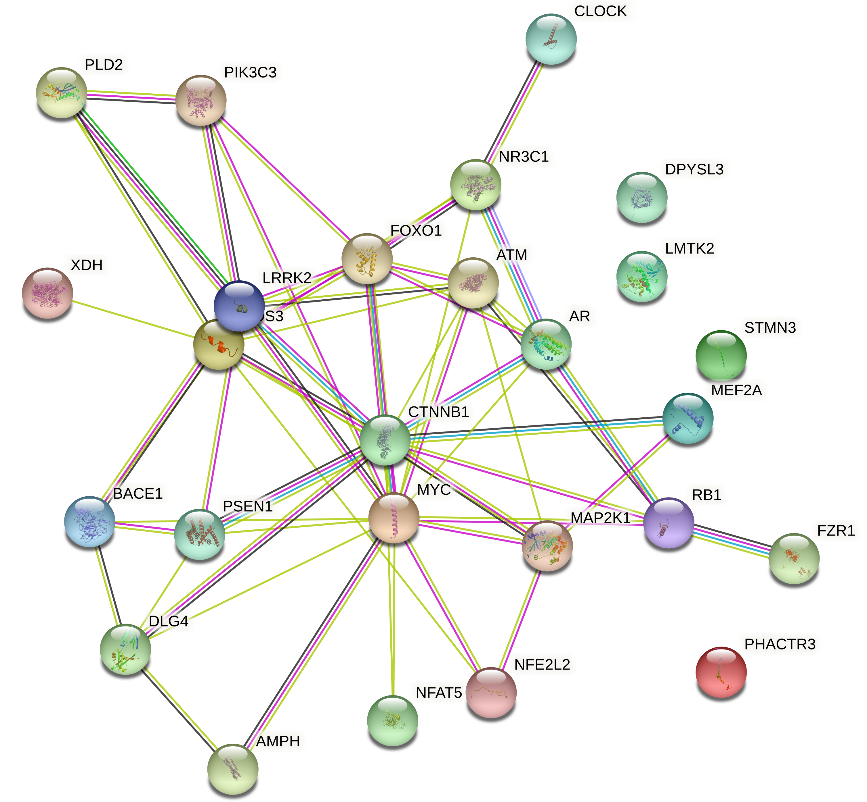


**Supplementary Figure S1.** Protein-protein interaction (PPI) network of CDK5 Kinase-target networks (STRING).


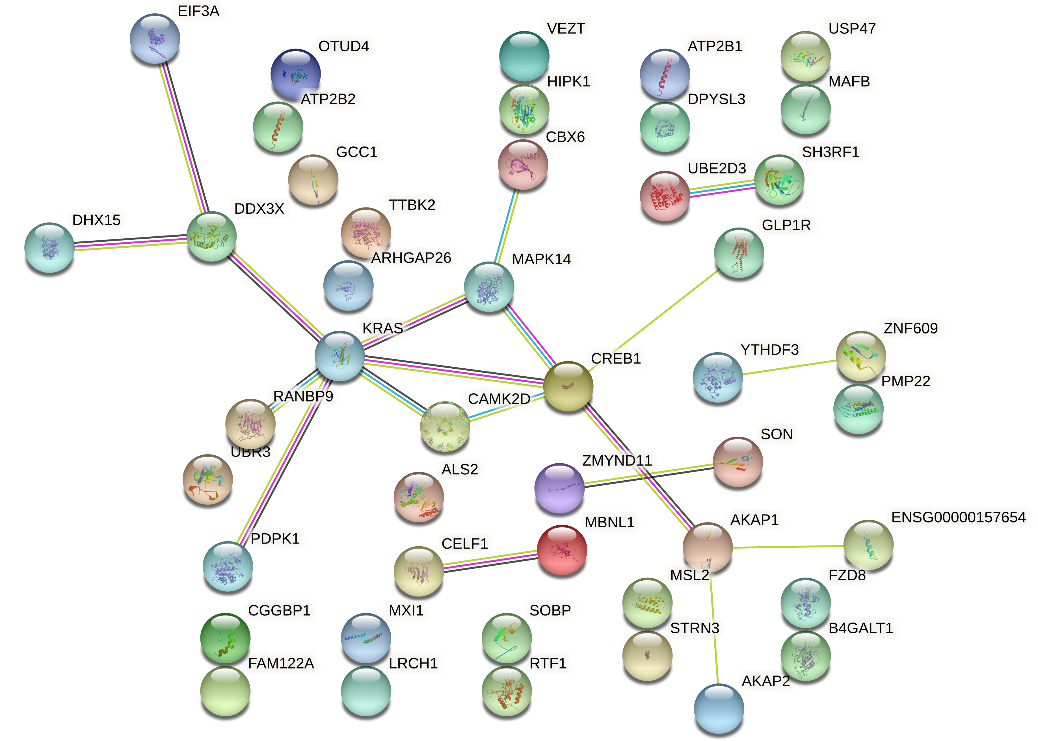


**Supplementary Figure S2.** PPI network of MIR-505 (STRING).
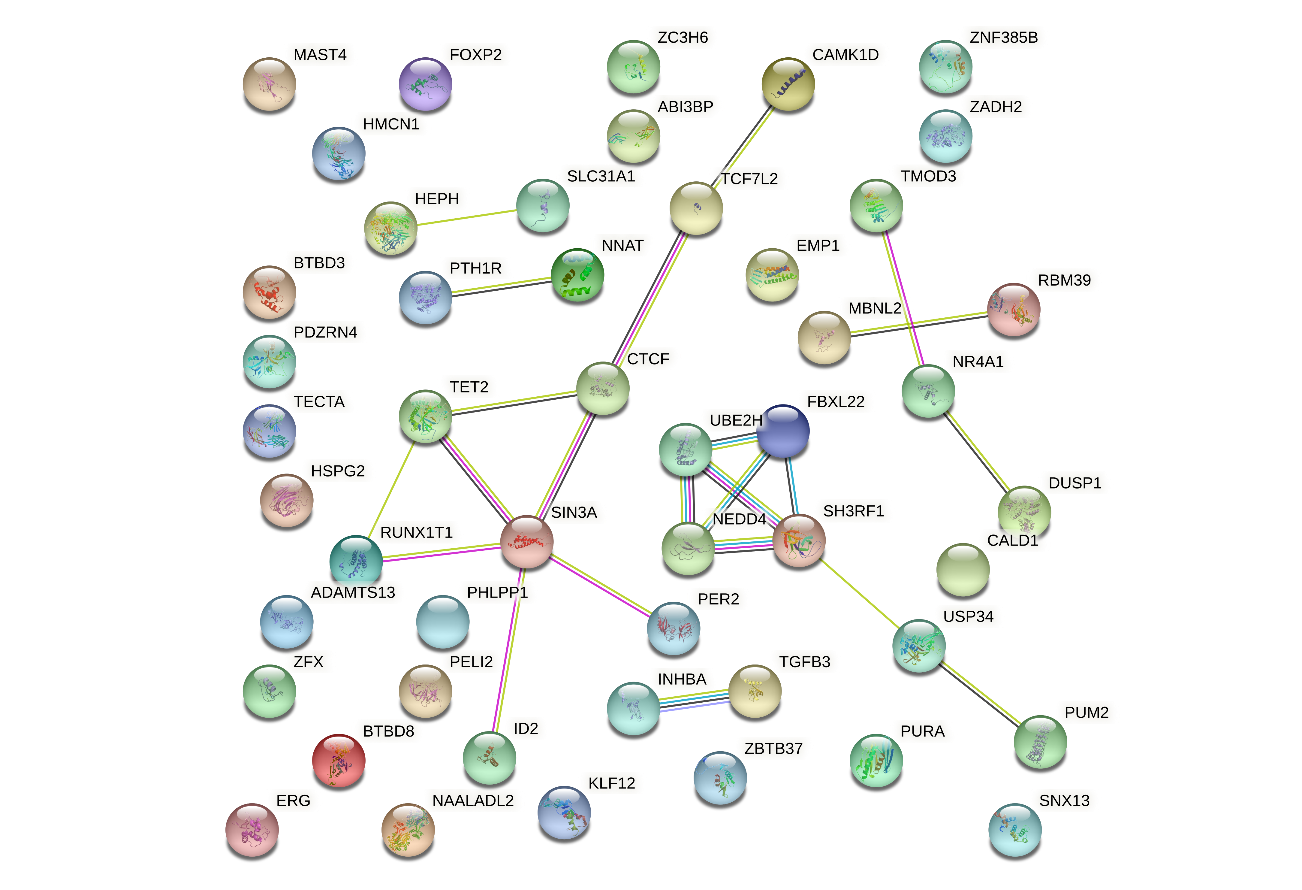


**Supplementary Figure S3.** PPI network of V$FREAC4_01 transcription factor -target networks (STRING).

## Supplementary Tables

**Supplementary Table S1.** Significantly enriched GO annotations (biological processes) of PDZD11 in LIHC (LinkedOmics)

| **Description** | **Leading Edge**  **Number** | **FDR** | **Leading Edge Gene** |
| --- | --- | --- | --- |
| mitochondrial respiratory chain complex assembly | 37 | 0 | COX17; NDUFA1; NDUFA11; NDUFA12; NDUFA13; NDUFA2; NDUFA3; NDUFA6; NDUFA7; NDUFA8; NDUFA9; NDUFAB1; NDUFAF2; NDUFAF3; NDUFAF4; NDUFB1; NDUFB10; NDUFB11; NDUFB2; NDUFB3; NDUFB4; NDUFB5; NDUFB6; NDUFB7; NDUFB9; NDUFC1; NDUFS3; NDUFS5; NDUFS6; NDUFS7; NDUFS8; NDUFV2; SDHAF2; SURF1; TAZ; UQCR10; UQCRB |
| translational elongation | 63 | 0 | AARS; ABTB1; AURKAIP1; CHCHD1; DAP3; DPH2; DPH3; EEF1D; EIF5A; EIF5AL1; ERAL1; GADD45GIP1; MRPL10; MRPL11; MRPL12; MRPL13; MRPL14; MRPL17; MRPL18; MRPL2; MRPL20; MRPL21; MRPL22; MRPL23; MRPL24; MRPL27; MRPL28; MRPL33; MRPL36; MRPL37; MRPL38; MRPL40; MRPL41; MRPL43; MRPL47; MRPL48; MRPL51; MRPL52; MRPL53; MRPL55; MRPL9; MRPS11; MRPS12; MRPS14; MRPS15; MRPS16; MRPS17; MRPS18A; MRPS21; MRPS23; MRPS24; MRPS26; MRPS30; MRPS33; MRPS34; MRPS36; MRPS5; MRPS7; MRPS9; RPLP1; RPLP2; TRNAU1AP; TUFM |
| nucleoside triphosphate metabolic process | 80 | 0 | ABCD1; ADA; AK1; AK2; ALDOA; BAD; BID; CCNB1; CDK1; CHCHD10; COX4I1; COX5A; COX5B; COX6A1; COX7A2; COX7C; CYC1; CYCS; DCTPP1; DGUOK; DTYMK; EIF6; ENO1; GAPDH; GPI; GUK1; IMPDH1; ITPA; NDUFA1; NDUFA12; NDUFA2; NDUFA3; NDUFA4; NDUFA6; NDUFA7; NDUFA8; NDUFA9; NDUFAB1; NDUFB1; NDUFB10; NDUFB2; NDUFB3; NDUFB4; NDUFB5; NDUFB6; NDUFB7; NDUFB9; NDUFC1; NDUFS3; NDUFS5; NDUFS6; NDUFS7; NDUFS8; NDUFV2; NME1; NME2; NME3; NME6; NUDT1; NUDT5; OLA1; PARK7; PFKFB4; PGK1; PRKAG1; RAN; RHOA; SDHAF2; SIRT6; SURF1; TAZ; TPI1; TSPO; UCK2; UQCR10; UQCRB; UQCRC1; UQCRH; UQCRHL; UQCRQ |
| proton transmembrane transport | 28 | 0 | ATP6AP1; ATP6V0B; ATP6V0C; ATP6V0D1; ATP6V0E1; ATP6V1C2; ATP6V1D; ATP6V1E1; ATP6V1E2; ATP6V1F; COX17; COX4I1; COX5A; COX5B; COX6A1; COX6B1; COX6C; COX7A2; COX7B; COX7B2; COX7C; COX8A; CYC1; NDUFA4; NDUFA4L2; PARK7; SLC25A14; SLC9A6 |

Abbreviations: FDR, false discovery rate from Benjamini and Hochberg from gene set enrichment analysis (GSEA).

**Supplementary Table S2.** Significantly enriched GO annotations (cellular components) of PDZD11 in LIHC (LinkedOmics)

| **Description** | **Leading Edge**  **Number** | **FDR** | **Leading Edge Gene** |
| --- | --- | --- | --- |
| mitochondrial protein complex | 108 | 0 | APOO; C12orf65; CHCHD1; CHCHD10; CHCHD3; CHCHD6; COX4I1; COX5A; COX5B; COX6A1; COX7A2; COX7C; CYC1; DAP3; HSD17B10; IMMP1L; MPV17L2; MRPL10; MRPL11; MRPL12; MRPL13; MRPL14; MRPL17; MRPL18; MRPL20; MRPL21; MRPL22; MRPL23; MRPL24; MRPL27; MRPL28; MRPL36; MRPL37; MRPL38; MRPL40; MRPL41; MRPL43; MRPL47; MRPL48; MRPL51; MRPL52; MRPL53; MRPL55; MRPL9; MRPS11; MRPS12; MRPS14; MRPS15; MRPS16; MRPS17; MRPS21; MRPS24; MRPS26; MRPS30; MRPS33; MRPS34; MRPS36; MRPS5; MRPS7; MRPS9; MTX1; NDUFA1; NDUFA11; NDUFA12; NDUFA13; NDUFA2; NDUFA3; NDUFA4; NDUFA4L2; NDUFA6; NDUFA7; NDUFA8; NDUFA9; NDUFAB1; NDUFB1; NDUFB10; NDUFB11; NDUFB2; NDUFB3; NDUFB4; NDUFB5; NDUFB6; NDUFB7; NDUFB9; NDUFC1; NDUFS3; NDUFS5; NDUFS6; NDUFS7; NDUFS8; NDUFV2; PARK7; ROMO1; TIMM10; TIMM17B; TIMM50; TIMM8B; TOMM40; TOMM5; TOMM6; TOMM7; UQCR10; UQCRB; UQCRC1; UQCRH; UQCRHL; UQCRQ; VDAC1 |
| respiratory chain | 47 | 0 | COX4I1; COX5A; COX5B; COX6A1; COX6B1; COX7A2; COX7B; COX7B2; COX7C; COX8A; CYC1; CYCS; HIGD2A; NDUFA1; NDUFA11; NDUFA12; NDUFA13; NDUFA2; NDUFA3; NDUFA4; NDUFA4L2; NDUFA6; NDUFA7; NDUFA8; NDUFA9; NDUFAB1; NDUFB1; NDUFB10; NDUFB11; NDUFB2; NDUFB3; NDUFB4; NDUFB6; NDUFB7; NDUFB9; NDUFC1; NDUFS3; NDUFS5; NDUFS6; NDUFS8; PARK7; UQCR10; UQCRB; UQCRC1; UQCRH; UQCRHL; UQCRQ |
| spliceosomal complex | 60 | 0 | BCAS2; BUD31; CWC15; CWC27; DDX41; EFTUD2; FRG1; GPKOW; HNRNPA3; HNRNPC; HTATSF1; IK; LSM2; LSM3; LSM4; LSM5; LSM7; MAGOH; MAGOHB; PPIE; PPIH; PRPF19; PRPF3; PRPF31; PRPF38A; PRPF6; RALY; RBM28; RBM3; RBM41; RHEB; RNF113A; SART1; SF3A2; SF3A3; SF3B4; SF3B5; SNRNP25; SNRNP35; SNRPA; SNRPA1; SNRPB; SNRPB2; SNRPC; SNRPD1; SNRPD2; SNRPD3; SNRPE; SNRPF; SNRPG; SYF2; TXNL4A; U2AF1L4; U2AF2; WDR83; YBX1; ZCRB1; ZMAT2; ZMAT5; ZRSR2 |

**Supplementary Table S3.** Significantly enriched GO annotations (molecular functions) of PDZD11 in LIHC (LinkedOmics)

| **Description** | **Leading Edge**  **Number** | **FDR** | **Leading Edge Gene** |
| --- | --- | --- | --- |
| structural constituent of ribosome | 100 | 0 | DAP3; MRPL10; MRPL11; MRPL12; MRPL13; MRPL14; MRPL17; MRPL18; MRPL2; MRPL20; MRPL21; MRPL22; MRPL23; MRPL24; MRPL27; MRPL28; MRPL33; MRPL34; MRPL36; MRPL37; MRPL41; MRPL43; MRPL47; MRPL51; MRPL52; MRPL55; MRPL9; MRPS11; MRPS12; MRPS14; MRPS15; MRPS16; MRPS17; MRPS18A; MRPS21; MRPS23; MRPS24; MRPS25; MRPS30; MRPS33; MRPS34; MRPS36; MRPS5; MRPS7; MRPS9; NDUFA7; RPL10; RPL10L; RPL11; RPL13; RPL14; RPL18; RPL19; RPL22; RPL23; RPL23A; RPL24; RPL26L1; RPL27; RPL27A; RPL28; RPL29; RPL30; RPL32; RPL35; RPL35A; RPL36; RPL36A; RPL36AL; RPL37; RPL37A; RPL38; RPL39; RPL5; RPL6; RPL8; RPLP1; RPLP2; RPS10; RPS11; RPS13; RPS14; RPS15; RPS15A; RPS16; RPS17; RPS19; RPS2; RPS20; RPS21; RPS23; RPS26; RPS27; RPS4X; RPS5; RPS7; RPS8; RPS9; RPSA; UBA52 |
| oxidoreductase activity, acting on NAD(P)H | 38 | 0 | AIFM1; AKR1C2; AKR1C3; CYB5R1; DHDH; NDUFA1; NDUFA12; NDUFA13; NDUFA2; NDUFA3; NDUFA4; NDUFA6; NDUFA7; NDUFA8; NDUFA9; NDUFAB1; NDUFAF2; NDUFB1; NDUFB10; NDUFB2; NDUFB3; NDUFB4; NDUFB5; NDUFB6; NDUFB7; NDUFB9; NDUFC1; NDUFS3; NDUFS5; NDUFS6; NDUFS7; NDUFS8; NDUFV2; NOX1; NQO1; NQO2; PGK1; TXNDC17 |
| oxidoreductase activity, acting on a heme group of donors | 14 | 0 | COX4I1; COX5A; COX5B; COX6A1; COX6B1; COX6C; COX7A2; COX7B; COX7B2; COX7C; COX8A; NDUFA4; NDUFA4L2; SURF1 |
| transmembrane receptor protein kinase activity | 47 | 0 | ACVR1; ACVR2A; ACVR2B; BMPR1A; BMPR2; CRIM1; DDR2; EFEMP1; EFNB3; EGFR; EPHA1; EPHA2; EPHA3; EPHA4; EPHA7; EPHB4; ERBB2; ERBB3; FGFR1; FGFR3; FGFRL1; FLT1; FLT3; FLT4; IGF1R; IGF2R; INSR; KDR; KIT; LMTK2; LTBP1; LTBP4; LTK; MERTK; MET; NRP1; NRP2; NTRK2; NTRK3; PDGFRA; PDGFRB; RET; ROR2; RYK; TEK; TGFBR2; TIE1 |

**Supplementary Table S4.** Significantly enriched KEGG pathway annotations of PDZD11 in LIHC (LinkedOmics)

| **Description** | **Leading Edge**  **Number** | **FDR** | **Leading Edge Gene** |
| --- | --- | --- | --- |
| Proteasome | 36 | 0 | ADRM1; POMP; PSMA1; PSMA3; PSMA4; PSMA5; PSMA6; PSMA7; PSMB1; PSMB10; PSMB2; PSMB3; PSMB4; PSMB5; PSMB6; PSMB7; PSMB8; PSMB9; PSMC1; PSMC2; PSMC3; PSMC4; PSMC5; PSMD1; PSMD11; PSMD12; PSMD13; PSMD14; PSMD2; PSMD3; PSMD4; PSMD6; PSMD7; PSMD8; PSME1; PSME2 |
| Ribosome | 85 | 0 | FAU; MRPL10; MRPL11; MRPL12; MRPL13; MRPL14; MRPL17; MRPL18; MRPL2; MRPL20; MRPL21; MRPL22; MRPL23; MRPL24; MRPL27; MRPL28; MRPL33; MRPL34; MRPL36; MRPL9; MRPS11; MRPS12; MRPS14; MRPS15; MRPS16; MRPS17; MRPS18A; MRPS21; MRPS5; MRPS7; MRPS9; RPL10; RPL10L; RPL11; RPL13; RPL14; RPL18; RPL19; RPL22; RPL23; RPL23A; RPL24; RPL26L1; RPL27; RPL27A; RPL28; RPL29; RPL30; RPL32; RPL35; RPL35A; RPL36; RPL36A; RPL36AL; RPL37; RPL37A; RPL38; RPL39; RPL5; RPL6; RPL8; RPLP1; RPLP2; RPS10; RPS11; RPS13; RPS14; RPS15; RPS15A; RPS16; RPS17; RPS19; RPS2; RPS20; RPS21; RPS23; RPS26; RPS27; RPS4X; RPS5; RPS7; RPS8; RPS9; RPSA; UBA52 |
| Non-alcoholic fatty liver disease (NAFLD) | 51 | 0 | CDC42; COX4I1; COX5A; COX5B; COX6A1; COX6B1; COX6C; COX7A2; COX7B; COX7B2; COX7C; COX8A; CYC1; CYCS; DDIT3; NDUFA1; NDUFA11; NDUFA12; NDUFA13; NDUFA2; NDUFA3; NDUFA4; NDUFA4L2; NDUFA6; NDUFA7; NDUFA8; NDUFA9; NDUFAB1; NDUFB1; NDUFB10; NDUFB11; NDUFB2; NDUFB3; NDUFB4; NDUFB6; NDUFB7; NDUFB9; NDUFC1; NDUFS3; NDUFS5; NDUFS6; NDUFS8; PRKAG1; RAC1; TRAF2; UQCR10; UQCRB; UQCRC1; UQCRH; UQCRHL; UQCRQ |
| Spliceosome | 48 | 0 | BCAS2; BUD31; CCDC12; CWC15; EFTUD2; HNRNPA3; HNRNPC; HSPA6; LSM2; LSM3; LSM4; LSM5; LSM7; MAGOH; MAGOHB; PPIE; PPIH; PQBP1; PRPF19; PRPF3; PRPF31; PRPF38A; PRPF6; PUF60; RBM22; RP9; SART1; SF3A2; SF3A3; SF3B4; SF3B5; SNRPA; SNRPA1; SNRPB; SNRPB2; SNRPC; SNRPD1; SNRPD2; SNRPD3; SNRPE; SNRPF; SNRPG; SYF2; THOC3; TXNL4A; U2AF1L4; U2AF2; ZMAT2 |
| DNA replication | 20 | 0 | FEN1; MCM2; MCM5; MCM6; MCM7; PCNA; POLA1; POLA2; POLD1; POLD4; POLE4; RFC2; RFC4; RNASEH1; RNASEH2A; RNASEH2B; RNASEH2C; RPA2; RPA3; SSBP1 |

**Supplementary Table S5.** Significantly enriched kinase-target networks of PDZD11 in LIHC (LinkedOmics)

| **Geneset** | **Leading Edge Gene** |
| --- | --- |
| Kinase_CDK5 | AMPH; AR; ATM; BACE1; CLOCK; CTNNB1; DLG4; DPYSL3; FOXO1; FZR1; LMTK2; LRRK2; MAP2K1; MEF2A; MYC; NFAT5; NFE2L2; NOS3; NR3C1; PHACTR3; PIK3C3; PLD2; PSEN1; RB1; STMN3; XDH |
| Kinase_NLK | CTNNB1; FOXO1; SMAD4; TCF4; TCF7L2 |
| Kinase_MAPK7 | DAPK1; DLG1; ELK4; ETS1; FOS; GJA1; MAP2K5; MEF2A; MEF2C; MYC; NFKB1; NR4A1; RPS6KA2; SGK1 |
| Kinase_DYRK1A | DYRK1A; FOXO1; LIN52; POLR2A; RCAN1; SF3B1; SPRY2 |

**Supplementary Table S6.** Significantly enriched miRNA-target networks of PDZD11 in LIHC (LinkedOmics)

| **Geneset** | **Leading Edge Gene** |
| --- | --- |
| GTGTTGA,MIR-505 | AKAP1; AKAP2; ALS2; ARHGAP26; ATP2B1; ATP2B2; B4GALT1; CAMK2D; CBX6; CELF1; CGGBP1; CREB1; DDX3X; DHX15; DPYSL3; EIF3A; FAM122A; FZD8; GCC1; GLP1R; HIPK1; KRAS; LRCH1; MAFB; MAPK14; MBNL1; MSL2; MXI1; OTUD4; PALM2-AKAP2; PDPK1; PMP22; RANBP9; RTF1; SH3RF1; SOBP; SON; STRN3; TTBK2; UBE2D3; UBR3; USP47; VEZT; YTHDF3; ZMYND11; ZNF609 |
| CAGTATT,MIR-200B,MIR-200C,MIR-429 | ACVR2A; ADIPOR2; AKAP2; AKAP7; AMBRA1; AMMECR1L; AMOTL2; ANKRD28; ARHGAP20; ARHGEF17; ARID2; ARID4B; ASXL1; ATP2A2; ATXN1; BAZ2B; BPTF; BRMS1L; BRWD1; CAB39; CACNA1C; CCNJ; CDK17; CDYL; CHD9; CLASP1; CLASP2; CLIP1; CNTN4; CRKL; CTDSPL2; DACH1; DACT1; DCUN1D1; DENND5A; DLC1; DUSP1; EFNB2; EGR3; ELF2; ETS1; EVI5; FAM76B; FAM8A1; FBXW11; FBXW2; FBXW7; FERMT2; FLI1; GABBR2; GABPA; GATA2; GEM; GLI3; GPM6A; HDHD2; HECTD2; HIC2; HIPK1; HLF; HMBOX1; HS3ST1; ID2; ITPR1; KANK1; KAT2B; KCNQ4; KIAA0355; KLF10; KLF12; LMO7; MAP4K3; MAP4K4; MATR3; MBLAC2; MED13; MEX3B; MEX3C; MGAT2; MPP5; MPRIP; MSL2; MTFR1; NAB1; NAP1L5; NBR1; NCOA2; NEGR1; NFYA; NGEF; NR3C1; NR5A2; NRIP1; NRP2; NTF3; NUDT4; NUFIP2; NUP153; OCLN; OSBPL11; OTUD4; OXR1; PALM2-AKAP2; PAPOLA; PCDH7; PDPK1; PDS5B; PHACTR3; PIKFYVE; PLCL1; PLK2; PPP1R10; PPP4R2; PUM2; QKI; R3HDM2; RANBP9; REV1; RLF; RND3; RPS6KA2; RTF1; SBF1; SDC2; SEC23A; SEMA6D; SERINC1; SESN1; SH3PXD2A; SLC16A12; SLC23A2; SLC38A4; SLC6A1; SMARCAD1; SMURF2; SYNJ1; TAF4; TEAD1; TOB1; TRIL; TRIM33; TSC22D1; TSC22D2; UBA6; USP25; VEZF1; WDFY3; YPEL2; YTHDF3; ZC3H6; ZCCHC24; ZEB1; ZEB2; ZFPM2; ZNF217 |
| ACTGAAA,MIR-30A-3P,MIR-30E-3P | AHCYL2; AKAP9; ANTXR2; AP1G1; AP4E1; APC; ARF6; ARID4A; ARPP19; ATXN1; BIRC6; C5orf24; CAPRIN1; CBL; CDC37L1; CDC40; COL12A1; CREBBP; CSNK1G3; DDX3X; DLG2; DLST; DNAJB14; DNAJB4; DNAJC27; DUSP8; EGR1; ELK3; EP300; EPAS1; EYA3; FAM13B; FBXW7; FCHO2; FGF7; FNDC5; GDF6; HERC4; HEXIM1; HIRA; KCNA3; KRAS; LRRTM2; MAPK6; MEF2C; MLLT10; NAA25; NEGR1; NHS; NR2C2; NR3C1; NUFIP2; OSBPL11; PAPOLG; PATZ1; PBRM1; PPP2R5E; PTEN; PURA; QKI; RARB; RICTOR; RUNX1T1; SEMA3C; SFRP1; SP3; SUN2; TMEM47; TNPO3; TNRC6B; TOB1; TOP1; TSC22D2; UBE2G1; VCPIP1; VGLL3; YOD1; ZEB2; ZFX; ZFYVE9; ZNF22 |
| AAAGGGA,MIR-204,MIR-211 | ADCY6; AKAP1; AP3M1; ARAP2; ARCN1; ARGLU1; ARHGAP29; ATF2; ATP2B1; BAZ2A; BCL2; BCL9L; BRD4; BRPF3; CAPRIN1; CCNT2; CCPG1; CDH2; CHN2; CLIP1; CRKL; DCAF5; DCUN1D3; DENND5A; DHH; DMTF1; DNAJC13; DTX1; DYRK1A; EDEM1; EFNB3; ELF2; ELL2; ELOVL6; EPHA7; ESR1; FAM160A2; FARP1; FBXW7; FNIP1; FRAS1; GAPVD1; GLIS3; GPM6A; HIC2; HOOK3; IGF2R; ITPR1; KCNA3; KDM2A; KLF12; LATS1; MAML3; MAP3K3; MBNL1; MED13L; MON2; MYO10; NBEA; NCOA7; NR3C1; NRBF2; NTRK2; PID1; PPP3R1; PRDM2; PRPF38B; RAB14; RHOBTB3; RHOT1; RICTOR; RPS6KA5; RSPO3; RUNX2; SATB2; SEC24D; SERINC3; SF3B1; SIN3A; SIRT1; SLTM; SMOC1; SOCS6; STXBP5; TAF5; TCF12; TGFBR2; TMOD3; TNRC6B; TRIP12; TTYH1; UHRF2; WEE1; XRN1; YTHDF3; ZCCHC14; ZCCHC24; ZDHHC17; ZFC3H1; ZFP91; ZNF423 |
| TACTTGA,MIR-26A,MIR-26B | ABHD2; ACADSB; ACBD5; ALDH5A1; ALS2; AMPH; ANKS1A; APC; ARID2; ARPP19; ASPN; ATF2; ATM; ATP1A2; BAZ2B; BCR; BHLHE40; BRWD1; BTBD7; CACNA1C; CAMSAP1; CD200; CDK6; CELSR1; CLASP2; COL10A1; CPSF2; CREBZF; CSNK1G1; CTTNBP2NL; DAPK1; DCDC2; DMXL1; EIF3A; EIF4G2; EIF5; ENC1; EP400; EPC2; EPHA2; ERLIN1; ESCO1; FBXO11; FNIP1; G3BP2; GSK3B; HAO1; HAS3; HERC4; HGF; JAG1; KALRN; KCNQ4; KLF10; LARP4; LGR4; LTBP1; MAN2A1; MAPK6; MED13L; MEX3B; MIB1; MRAS; MXI1; NAB1; NAP1L5; NDFIP2; NHS; NRIP1; NTN4; NUP50; OSBPL11; OTUD4; PALMD; PAN3; PATZ1; PAWR; PCDH18; PCK1; PDGFRA; PDHX; PELI2; PFKFB3; PHLDB2; PLP1; POM121; PPP3R1; PSD3; PTGS2; PTP4A1; PTPN13; PURA; RANBP10; RGS4; RLF; RNF6; RPS6KA2; RTF1; SALL1; SH3D19; SH3PXD2A; SLC12A2; SLC22A23; SLC25A16; SLC38A2; SLC4A4; SMAD1; SMAD4; STRADB; STYX; TBC1D4; TNRC6A; TNRC6B; TOB1; UBE2G1; UBR3; ULK2; USP15; USP25; USP3; VANGL2; WNK3; YPEL1; YTHDF3; ZCCHC24; ZDHHC6; ZFHX4; ZFX; ZNF217; ZNF462; ZNF608 |

**Supplementary Table S7.** Significantly enriched transcription factor-target networks of PDZD11 in LIHC (LinkedOmics)

| **Geneset** | **Leading Edge Gene** |
| --- | --- |
| GGAANCGGAANY_UNKNOWN | ATP6V1D; ATP6V1E1; BANF1; CHMP2A; COX6B1; COX7A2; CSNK2B; EBNA1BP2; EIF2S3; EIF3K; MED8; MRPL21; MRPL43; MRPS21; MRPS23; PDAP1; POMP; PRPF3; PSMB4; RARS; RNF25; RPL38; RRAS; RUVBL2; SEC61A1; SEC61G; SMUG1; SNRPE; TAF10; TIMM8A; TMCO1; UBA52; UBL5; UBXN1; VPS16 |
| V$FREAC4_01 | ABI3BP; ADAMTS13; BTBD3; BTBD8; CALD1; CAMK1D; CTCF; DUSP1; EMP1; ERG; FBXL22; FOXP2; HEPH; HMCN1; HSPG2; ID2; INHBA; KLF12; MAST4; MBNL2; NAALADL2; NEDD4; NNAT; NR4A1; PDZRN4; PELI2; PER2; PHLPP1; PTH1R; PUM2; PURA; RBM39; RUNX1T1; SH3RF1; SIN3A; SLC31A1; SNX13; TCF7L2; TECTA; TET2; TGFB3; TMOD3; UBE2H; USP34; ZADH2; ZBTB37; ZC3H6; ZFX; ZNF385B |
| V$HOX13_01 | AMMECR1L; CALD1; COLEC10; EHBP1; FAM135B; FOXA1; NEO1; NTF3; PDE2A; SMOC1; SON; SVIL; TNRC6A; UNC5C; YES1 |
| V$SOX9_B1 | ACVR2A; APBA1; BCL2L11; BPTF; CADPS; CCNJ; CDK8; CHD6; CSDC2; CTNNB1; CYB5D1; DAAM1; DDX6; DENND2C; DLC1; DMD; DOCK4; DTX1; DUSP6; EGFLAM; EHBP1; ERG; FAM122A; FBLN5; FLRT3; G2E3; G3BP2; GNAO1; HHEX; HSPG2; INHBA; KLF3; LARP4; LHX6; MAML1; MAP3K3; MAP4K4; MBP; MLLT6; MYCT1; MYO18A; N4BP1; NAV1; NEO1; NFIX; NGFR; NR4A1; OLFM1; PBX1; PCDH12; PDS5B; PELI2; PICALM; PPP1R16B; PRKCH; PUM1; RAB5A; RBM39; RREB1; SASH1; SLC13A5; SLC39A14; SLC39A9; SLIT2; SORBS2; SOX5; SPTBN1; STK38L; SYTL2; TAL1; TEAD1; TEK; TIA1; TMEM88; TOP1; TSPAN2; UBE3A; USP25; WEE1; ZFYVE1 |
| V$STAT5A_02 | ASXL1; ATOH8; BCL6; BMP5; BTRC; CDC37L1; CHD2; DCUN1D3; DDX17; DDX25; DSG1; EMP1; FOS; FOXA3; GABPA; GJA5; GRAMD1C; HNF4G; IL1RN; KCNJ8; KLF9; LHX6; LRRN3; MAT1A; MBNL1; MBNL3; MGRN1; NCKAP5; NFATC2; NR4A3; PATZ1; PCSK2; PDK4; PIK3R1; PILRB; PPP1R16B; PTCH1; PTPN21; SCN2A; SETD2; SOCS2; SPTBN2; SRPX; TAL1; TRAF6; ZFR; ZNF180; ZNF184; ZNF423; ZNF689 |
